# Polygenic heterogeneity in antidepressant treatment and placebo response

**DOI:** 10.1038/s41398-022-02221-4

**Published:** 2022-10-29

**Authors:** Anne Krogh Nøhr, Annika Forsingdal, Ida Moltke, Oliver D. Howes, Morana Vitezic, Anders Albrechtsen, Maria Dalby

**Affiliations:** 1 The Bioinformatics Centre, Department of Biology, University of Copenhagen, Copenhagen N, Denmark; 2 H. Lundbeck A/S, Valby, Copenhagen, Denmark; 3 H. Lundbeck A/S, St Albans, UK; 4 IoPPN, King’s College London and Imperial College London, London, UK; 5 Institute of Clinical Sciences, Faculty of Medicine, Imperial College London, London, UK

**Keywords:** Predictive markers, Depression

## Abstract

The genetic architecture of antidepressant response is poorly understood. Polygenic risk scores (PRS), exploration of placebo response and the use of sub-scales might provide insights. Here, we investigate the association between PRSs for relevant complex traits and response to vortioxetine treatment and placebo using clinical scales, including sub-scales and self-reported assessments. We collected a clinical test sample of Major Depressive Disorder (MDD) patients treated with vortioxetine (*N* = 907) or placebo (*N* = 455) from seven randomized, double-blind, clinical trials. In parallel, we obtained data from an observational web-based study of vortioxetine-treated patients (*N* = 642) with self-reported response. PRSs for antidepressant response, psychiatric disorders, and symptom traits were derived using summary statistics from well-powered genome-wide association studies (GWAS). Association tests were performed between the PRSs and treatment response in each of the two test samples and empirical *p*-values were evaluated. In the clinical test sample, no PRSs were significantly associated with response to vortioxetine treatment or placebo following Bonferroni correction. However, clinically assessed treatment response PRS was nominally associated with vortioxetine treatment and placebo response given by several secondary outcome scales (improvement on HAM-A, HAM-A Psychic Anxiety sub-scale, CPFQ & PDQ), (*P* ≤ 0.026). Further, higher subjective well-being PRS (*P* ≤ 0.033) and lower depression PRS (*P* = 0.01) were nominally associated with higher placebo response. In the self-reported test sample, higher schizophrenia PRS was significantly associated with poorer self-reported response (*P* = 0.0001). The identified PRSs explain a low proportion of the variance (1.2–5.3%) in placebo and treatment response. Although the results were limited, we believe that PRS associations bear unredeemed potential as a predictor for treatment response, as more well-powered and phenotypically similar GWAS bases become available.

## Introduction

Second-generation antidepressants, such as selective serotonin reuptake inhibitors (SSRIs), are the first-line treatment for Major Depressive Disorder (MDD). Although these drugs demonstrate greater efficacy over placebo, around 50% of patients do not achieve remission and more than 30% do not respond during the first 6–12 weeks of treatment [1, 2]. For non-responding patients, the process of finding an effective treatment can be long, delaying recovery and imposing a high burden for the individual and to society [3, 4]. With MDD representing the leading cause of disability worldwide and exhibiting a growing prevalence in the general population [5–9] it is critical to develop more personalized treatment options to improve outcomes.

Based on dense single-nucleotide polymorphism (SNP) chip data of 5151 individuals, the SNP heritability for clinically assessed remission in MDD has been estimated to 13.2% [10]. However, no variants predictive of antidepressant response have been robustly replicated from candidate gene studies [11] or genome-wide association studies (GWAS) [10, 12]. This is most likely due to the limited sample sizes available, the variation in clinical trial designs, including various drugs, patient diagnosis and clinical endpoints, and the complex polygenic architecture of treatment response.

Instead of focusing on individual variants that predict antidepressant response, another approach is to combine information across genetic loci through polygenic risk scores (PRS), the weighted sum of risk alleles associated with a trait for a given individual [13]. A few recent studies have explored the association between antidepressant treatment response and PRSs for psychiatric disorders and personality traits. A higher attention-deficit hyperactivity disorder (ADHD) PRS was associated with higher risk of treatment resistant depression [14]. Furthermore, a higher PRS for MDD [12, 14–16], schizophrenia [12, 16, 17] and neuroticism [14–16] have shown nominal associations with worse treatment response or antidepressant treatment resistance. A recent study, presented the largest GWAS of clinically assessed antidepressant response [10]. Using an independent cohort, the study found a nominally significant association for antidepressant response and PRS for antidepressant remission calculated from their own GWAS summary statistics. Furthermore, they have released this valuable data resource for others to use.

A limitation of the current literature is that placebo response has not yet been studied in a PRS context. This is essential to be able to develop genetic predictors of response to a given treatment, rather than non-specific response, and necessary to personalize treatment decisions. Thus, investigating the genetics of placebo response in MDD could identify potential differences and similarities in the genetic underpinnings of drug efficacy and placebo response. A further question is whether there are genetic predictors of response within specific symptom domains of depression severity, such as anxiety and cognitive deficits, which could be used to further personalize treatment.

Motivated by this, the aim of this study was to assess PRSs associations with placebo and antidepressant treatment response from pooled clinical trial data and test its generalizability to real-world data from a web-based survey conducted by 23andMe Inc. [18]. We use a deep clinical test sample (*N* = 1364) with vortioxetine and placebo-treated patients and a self-reported test sample with vortioxetine-treated patients (*N* = 642). Vortioxetine is an antidepressant approved for treatment of MDD, which in addition to the antidepressant effect also has an anxiolytic effect and reduces cognitive impairment [19]. In the clinical test sample, vortioxetine and placebo response were measured by several clinical scales and sub-scales in patients with MDD from seven randomized, double-blind, placebo-controlled clinical trials. Using both test samples and taking advantage of the recently published GWAS summary statistics of antidepressant treatment response (self-reported responders, clinically assessed response), psychiatric disorder (ADHD, bipolar, schizophrenia and MDD), and symptom traits (subjective well-being, neuroticism, and cognition), we investigate the following questions: (1) Is PRS for self-reported treatment responders or clinically assessed treatment response associated with vortioxetine response in our self-reported and clinical test samples? (2) Is a treatment response PRS more associated with vortioxetine response than a PRS for disease or symptom trait? (3) Is PRS for treatment response, disease, or symptom trait associated with placebo response in the clinical test sample, and how does it compare to PRS associations observed for vortioxetine response?

## Methods and materials

### Clinical test sample

#### Patients and trial design

The clinical test sample included MDD patients from seven randomized, double-blind, placebo-controlled clinical trials evaluating the efficacy of vortioxetine, see Table 1. The patients were treated for 6 or 8 weeks with vortioxetine (2.5, 5, 10, 15, or 20 mg/day), venlafaxine XL (25 mg/day), duloxetine (60 mg/day) or placebo. Patients were clinically diagnosed with MDD by DSM-IV-TR [20]. They were currently in a depressive episode of ≥3 months’ duration and had a Montgomery-Åsberg Depression Rating Scale [21] (MADRS) total score ≥26 at the baseline visit. Among the shared exclusion criteria for all 7 clinical trials were current psychiatric disorder other than MDD, taking psychotropic drugs within two weeks prior to baseline or during the study and significant risk of suicidality.

**Table 1:** Characteristics of randomized, double-blind, placebo-controlled clinical trials included in the clinical test sample.

ClinicalTrials.gov Identifier	Treatment length, weeks	Vortioxetine dose, mg	Scales	*N*	Female, %	Age, mean (SD)	Baseline MADRS, mean (SD)
NCT01422213	8	10, 20	MADRS, PDQ	184	63.04	46.22 (11.98)	31.75 (3.77)
NCT00839423	6	5,10	MADRS, HAM-A	136	66.18	42.42 (12.04)	33.74 (2.57)
NCT00635219	8	2.5, 5, 10	MADRS, HAM-A	254	70.08	45.70 (12.20)	31.69 (3.60)
NCT01140906	8	15, 20	MADRS, HAM-A	233	64.81	46.79 (13.60)	31.23 (3.34)
NCT01153009	8	15, 20	MADRS, HAM-A	196	77.04	43.77 (12.71)	31.92 (4.20)
NCT01163266	8	10, 20	MADRS, HAM-A, CPFQ	178	74.72	43.42 (12.77)	32.05 (3.78)
NCT01179516	8	10, 15	MADRS, HAM-A, CPFQ	183	69.95	45.95 (12.30)	33.59 (4.03)
Total	6–8	2.5, 5, 10, 15, 20	MADRS, PDQ, HAM-A, CPFQ	1364	69.43	45.09 (12.62)	32.16 (3.76)

The table only include patients who passed QC and was used in later analysis.

*MADRS* Montgomery-Åsberg Depression Rating Scale, *HAM-A* Hamilton Anxiety Rating Scale, *PDQ* perceived deficits questionnaire, *CPFQ* Massachusetts General Hospital cognitive and physical functioning questionnaire, *SD* standard deviation, *baseline MADRS* Total MADRS score at baseline.

This research was conducted in accordance with the Declaration of Helsinki and used pseudo-anonymous human biological material and clinical data. Prior to participation all patients provided written, informed consent to participate, including consent for exploratory genetic research.

#### Measures of antidepressant response

In the clinical test sample, treatment response was assessed using one primary and five secondary response outcomes. The primary outcome was the total improvement from baseline (change from baseline * (−1)) in depression symptoms measured by MADRS [21]. The outcome was quantified as an improvement, so that a higher value means better response. The secondary outcomes were total improvement from baseline in; depression symptoms measured by the sub-scale MADRS-6; anxiety symptoms measured by Hamilton Anxiety Rating Scale (HAM-A) [22] and sub-scales HAM-A Psychic Anxiety (PA) and HAM-A Somatic Anxiety (SA). The last secondary outcome was relative improvement from baseline (relative change from baseline * (−1)) in cognitive dysfunction measured by the Perceived deficits questionnaire (PDQ) [23] or Massachusetts General Hospital cognitive and physical functioning questionnaire (CPFQ) [24]. Thus, vortioxetine and placebo response are referred to as MADRS improvement, MADRS-6 improvement, HAM-A improvement, HAM-A PA improvement, HAM-A SA improvement and PDQ & CPFQ improvement. A detailed description of the scales and sub-scales are provided in Supplementary Material A.

**Fig. 1:**
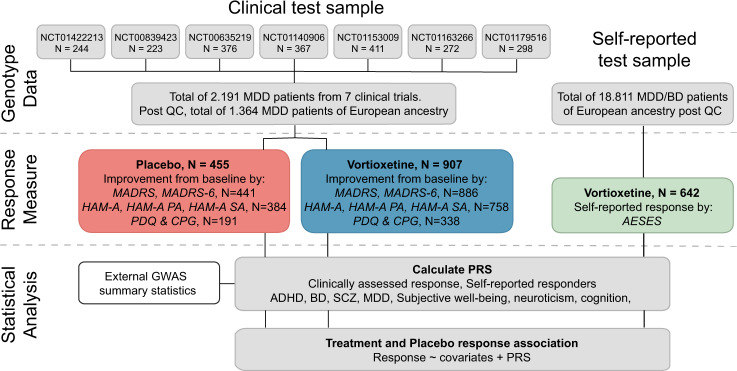
Overview of the samples and study design. The clinical test sample included 2191 genotyped MDD patients treated with vortioxetine or placebo from 7 randomized, double-blind, placebo-controlled clinical trials. 907 vortioxetine-treated patients and 455 placebo-treated patients of European genetic ancestry passed genotyping quality control (QC). Response was quantified using different clinical scales and sub-scales, resulting in 6 response measures for both the vortioxetine and the placebo group. The self-reported test sample of European ancestry participants suffering from depression or bipolar disorder identified 642 vortioxetine-treated participants based on the AESES questionnaire. In the statistical analysis, PRSs for treatment response, diseases, and symptom traits were calculated for everyone in both samples. Vortioxetine and placebo response association with these PRSs were estimated.

Patients were treated for 8 weeks, except in study NCT00839423 which lasted 6 weeks. As it takes approximately 4 weeks from the start of treatment until a clinician can reliably detect whether a treatment is working [25], only patients with a minimum of 4 weeks of treatment were included in the analysis. For patients with missing data, the last observation carried forward method was used. Figure 1 provides a flow diagram of the method.

#### SNP genotyping and quality control (QC)

In the clinical test sample, blood samples were collected at baseline and stored in the Lundbeck Biobank. Samples from 2191 individuals were genotyped in three batches using Illumina Omni5Exome4 v1.1 SNP arrays. Genotype calls by clustering were performed using GenomeStudio (v2011.1).

Basic QC was conducted in PLINK 2.0 [26]. First, each genotype batch underwent QC including removal of individuals with missingness below 0.02 and the conversion of all SNPs to the hg19 forward strand. Even after converting all SNPs to the forward strand, we observed flipping errors when merging with 1000 Genomes Phase 3 reference panel. Therefore, SNPs were removed if they were not in the reference panel and if their allele frequency had an absolute difference from the reference panel greater than 0.25. Finally, the three batches were merged and only individuals with a European ancestry fraction > 95% were kept, identified using ADMIXTURE [27].

We then conducted further QC of the sample to remove SNPs with one or more of the following: minor allele frequency (MAF) < 0.01, missingness > 0.02, Hardy–Weinberg (HWE) *p*-value < 0.00001. We removed individuals with missingness > 0.02, that failed relatedness check given by one individual from a pair with IBD > 0.25 or *k*1 = > 0.3 or *k*2 = > 0.3, who were > 6 standard deviations (SD) away from the mean of each of the principal components (PCs) calculated using Eigensoft [28], or who had a heterozygosity score > 4 SD from the mean. In total, 2.166.166 SNPs and 1.364 individuals of European ancestry passed QC. The restriction to European ancestry accounted for the greatest loss of individuals. PCs used as covariates in subsequent analysis were based on genotyped SNPs and calculated using Eigensoft.

The data was imputed to the 1000 Genomes Phase 3 reference panel using the Docker version of the Michigan Imputation Server [29]. Post imputation QC using PLINK 2.0 removed SNPs with MAF < 0.01, missingness > 0.02, imputation score (*R* [2]) < = 0.3, duplicated and non-autosomal SNPs. A final set of 8,316,717 SNPs were included for analysis.

In the clinical test sample, genome-wide association testing was performed using Plink v2 for each of the six measures of placebo and vortioxetine response using a linear model and adjusting for sex, age and PC1-PC8. Dose was also included as a covariate in the vortioxetine response group. The generated summary statistics were used to calculate SNP heritability and genetic correlations for the six placebo and vortioxetine response outcomes using LD Score regression (LDSC) [30]. SNP heritability was also calculated from individual-level data using GREML in the genome-wide complex trait analysis (GCTA) [31] software. More details are provided in Supplementary Material B.

### Self-reported test sample

The self-reported sample was obtained from an observational 23andMe Inc. study, named the AFFECT study [18] of 18,811 European participants with a self-reported diagnosis of depression or bipolar disorder by a medical professional and medication status data. The study included 642 study participants of European genetic ancestry who had received vortioxetine within the past five years and had rated the response from 0 (not at all) to 4 (a great deal). The participants had a diagnosis of either MDD (*N* = 419) or bipolar disorder (*N* = 254) and were US citizens. Samples were genotyped, phased, and imputed by 23andMe standardized pipeline and vortioxetine treatment response were obtained as part of the Antidepressant Efficacy and Side Effects (AESES) questionnaire [32]. Roughly 9.01 million high-quality genotyped and imputed SNPs on autosomal and X chromosomes were used in this analysis.

### Base GWAS summary statistics QC

Publicly available summary statistics from GWAS of nine different phenotypes were used as bases for calculating the PRSs used in this study. The phenotypes were self-reported treatment responders [17], clinically assessed treatment response (percentage improvement) [10], MDD [33], bipolar disorder [34], schizophrenia [35], ADHD [36], subjective well-being [37], neuroticism [38], and general cognitive function [39]. An overview of the base summary statistics is provided in Supplementary Material Table S1. QC was performed by removal of; SNPs with MAF < 1% and INFO < 0.8, SNPs within the MHC region, duplicate, and ambiguous SNPs.

### Polygenic risk scores in clinical test sample

Polygenic risk scores (PRSs) were calculated using an additive model for the nine base GWAS summary statistics using PRSice v2.3.5 [40]. The PRSs were standardized to have zero mean and a standard deviation of one. Clumping with window = 250 kb and *r*^2^ = 0.1 was performed to remove SNPs in LD. PRSs were constructed for 11 *p*-value thresholds (pTs) in the range of 0.000001 to 1. In the clinical test sample, the association between the PRSs for each pT and the 6 vortioxetine and placebo response measures were evaluated using linear regression and adjusted for sex, age and PC1-PC8. The dose was also included as a covariate for patients treated with vortioxetine. We observed no significant correlations between baseline symptom severity and any of the outcome measures (see Supplementary Materials B). Therefore, baseline symptom severity was not included as a covariate in the PRS association analyses. The pT resulting in the most predictive PRS was identified. We controlled for type one errors by calculating an empirical p-value for the most predictive PRS using 10000 permutations. We conducted a total of 108 tests (2*(treatment group)*9(base GWAS)*6(response outcome measure)) and controlled for multiple testing using Bonferroni correction (0.05/108 = 0.00046). When describing results from this study, nominally significance means empirical *p*-values < 0.05 that did not reach the Bonferroni significance threshold.

### Polygenic risk scores in self-reported sample

PRS analysis in the self-reported sample was conducted as described above. Association tests between the self-reported vortioxetine response measure and PRSs of the nine base GWAS summary statistics for 11 pTs were conducted while adjusted for sex, age, genotype array, diagnosis and five PCs. PRS was also performed using a 10,000 permutation test and empirical *p*-values were reported. Since the self-reported test sample only includes vortioxetine-treated patients and one phenotype, the significance threshold after Bonferroni correction was 0.0056 (0.05/9 = 0.0056).

## Results

### Test sample characteristics

The clinical test sample consists of 1364 depressed patients of European genetic ancestry. 907 were treated with vortioxetine and 457 received placebo. Demographics and baseline characteristics are provided in Table 1 and in Supplementary Material Table S4. The clinical test sample was 69.4 % female with a mean age of 45.1 (SD = 12.6) years. Patients were moderately to severely depressed, with a mean baseline MADRS score of 32.2 (SD = 3.8). At baseline there were no clinically relevant differences between placebo and vortioxetine in demographic or clinical characteristics after pooling the studies. Overall, patients treated with vortioxetine showed a greater average improvement from baseline on all scales and sub-scales compared to the placebo treated patients (Supplementary Material Table S5).

The self-reported test sample included 642 study participants of European genetic ancestry who had received vortioxetine within the past five years. The sample was 80.8 % female and age ranged from 18–50 years (mean (SD) = 35.2 (7.9)). Study participants were moderately depressed, with a mean baseline PROMIS depression T-score [21, 41] of 64.1 (SD = 8.61).

Expectedly, no significant genome-wide-associations, SNP-based heritability, or genetic correlations for vortioxetine response or placebo response were observed, see Supplementary Material B and Supplementary Material Figs. S1–S4.

### Treatment response PRSs association with vortioxetine response

We investigated whether PRS for self-reported treatment responders and clinically assessed treatment response was associated with vortioxetine response in our clinical and self-reported test samples. PRS associations with an empirical *p*-value below 0.05 are listed in Table 2. All PRS associations are listed in Supplementary Material Table C and visualized in Supplementary Material Figs. S8, S9. Power to conduct PRS analysis was explored for different sample sizes of our test samples to evaluate our results, see Supplementary Material B.

**Table 2:** Associations with an empirical *p*-value < 0.05 between PRS for antidepressant response, psychiatric disorders, and symptom traits and placebo and vortioxetine response in the clinical test sample and the self-reported test sample.

PRS	Response measure	*N*	Best P_T_	N SNP	PRS *R*^2^	Full *R*^2^	Beta	SE	Emp. p
*Clinical test sample—vortioxetine response*									
Clinically assessed response	HAM-A improvement	758	0.0001	87	0.012	0.122	0.82	0.28	0.026
Clinically assessed response	HAM-A PA improvement	758	0.0001	87	0.014	0.080	0.58	0.18	0.014
*Clinical test sample—placebo response*									
MDD	HAM-A SA improvement	384	0.00005	730	0.022	0.056	0.54	0.19	0.011
Clinically assessed response	CPFQ & PDQ improvement	191	0.1	31466	0.053	0.088	4.82	1.54	0.004
Subjective well-being	MADRS improvement	441	0.001	834	0.019	0.043	−1.45	0.50	0.031
Subjective well-being	MADRS-6 improvement	441	0.001	834	0.019	0.042	−0.97	0.34	0.033
Subjective well-being	HAM-A SA improvement	384	0.005	2806	0.023	0.056	−0.57	0.20	0.028
*Self-reported test sample—vortioxetine response*									
Schizophrenia	Self-reported response	742	0.0001	2325	0.036	0.048	−0.283	0.058	0.0001*

*PRS* polygenic risk score, *MDD* major depressive disorder, *MADRS* Montgomery-Åsberg Depression Rating Scale, *MADRS-6* sub-scale of MADRS that focuses on the core symptoms of depression, *HAM-A* Hamilton Anxiety Rating Scale, *HAM-A SA* sub-scale of HAM-A that focuses on somatic anxiety, *HAM-A PA* sub-scale of HAM-A that focuses on psychic anxiety, *PDQ* Perceived deficits questionnaire, *CPFQ* Massachusetts General Hospital cognitive and physical functioning questionnaire, *N* no. of patients in association test, *Best P*_*T*_ Best *P*-value threshold as defined pr PRSice2, *N SNP* no. of SNPs for bets P_T_, *PRS R*^*2*^ variance explained by the PRS, *Full R*^*2*^ variance explained by the full model (including covariates), *Beta* estimated coefficient, *SE* standard error, *P* P-value, *Emp. p* Empirical *p*-values.

*Significant following Bonferroni correction.

**Fig. 2:**
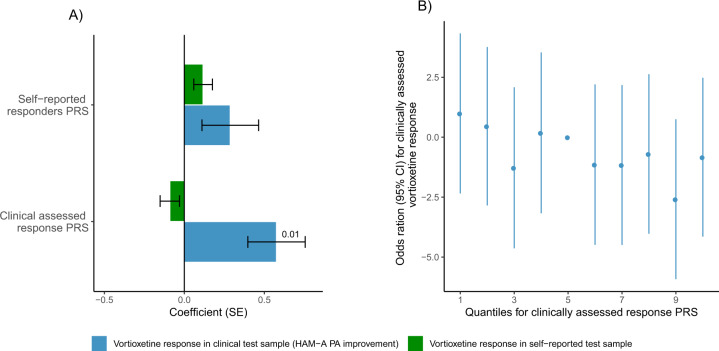
Treatment response PRSs association with vortioxetine response. **A** Bar plot showing the coefficients of self-reported responders PRS and clinically assessed response PRS association with vortioxetine response in the clinical and self-reported test samples. **B** Odds ratios for vortioxetine response given by HAM-A PA improvement in the clinical test sample for different quantiles of clinically assessed response PRS.

For patients treated with vortioxetine in the clinical test sample no associations were significant after multiple testing. Higher clinically assessed response PRS was nominally associated with improvement in anxiety symptoms (HAM-A improvement: pT = 0.0001, beta (SE) = 0.82(0.28), emp. *p*-value = 0.026 and HAM-A PA improvement: pT = 0.0001, beta (SE) = 0.58 (0.18), emp. *p*-value = 0.014), see Fig. 2A, B. All six response measures assessing vortioxetine response in the clinical test sample showed nominal associations of the same direction, see Fig. 4A. In the self-reported test sample, neither a self-reported treatment responders PRS nor a clinically assessed treatment response PRS was associated with self-reported vortioxetine treatment response.

### Disease and symptom trait PRSs association with vortioxetine response

Next, we examined whether PRSs for a disease and symptom trait were associated with treatment response. In the clinical and self-reported test samples, PRS for ADHD, bipolar disorder, schizophrenia, MDD, subjective well-being, neuroticism and cognition was tested for associations with vortioxetine response.

**Fig. 3:**
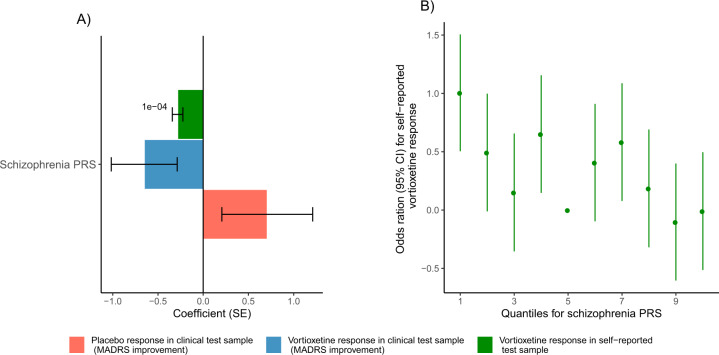
Schizophrenia PRS association with vortioxetine response. **A** Bar plot showing the coefficients of schizophrenia PRS association with placebo and vortioxetine response in the clinical and self-reported test sample. **B** Odds ratios for vortioxetine response in the self-reported sample for quantiles of schizophrenia PRS.

In the self-reported test sample, schizophrenia PRS was significantly associated with self-reported vortioxetine response (pT = 0.0001, beta (SE) = −0.28 (0.06), emp. *p*-value = 0.0001), where higher schizophrenia PRS was associated with worse vortioxetine response, see Fig. 3A, B. In the clinical test sample, no associations with an empirical *p* value < 0.05 were observed. However, in the clinical test sample we observed effect sizes in the same direction as the previous finding, where higher schizophrenia PRS were associated with worse vortioxetine response measured by MADRS improvement (pT = 1, beta (SE) = −0.55 (0.36), emp. *p*-value = 0.39) and MADRS-6 improvement (pT = 0.001, beta (SE) = −0.41 (0.24), emp. *p*-value = 0.31), see Fig. 3A.

### Treatment response, disease, and symptom trait PRSs association with placebo response

Finally, we investigated whether PRSs for relevant complex traits were associated with placebo response and how potential associations compare to the associations observed for vortioxetine response in the clinical test sample.

**Fig. 4:**
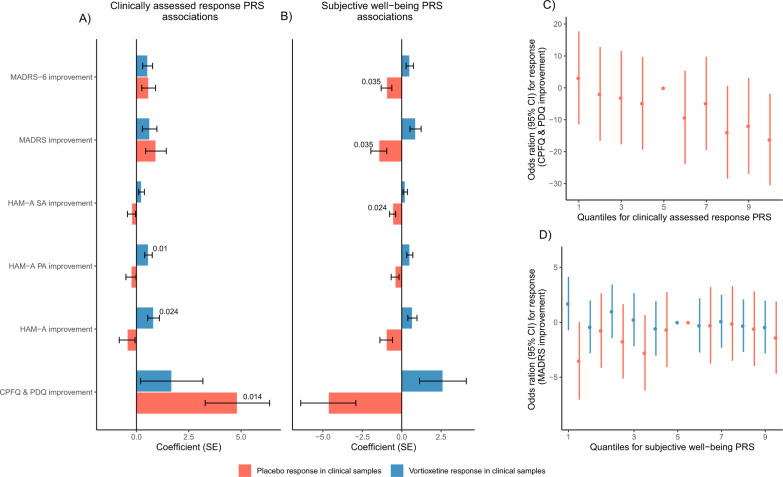
Clinically assessed response and subjective well-being PRSs association with placebo response. Bar plot showing the coefficients for all placebo and vortioxetine response outcome measures in the clinical test sample association with **A** clinically assessed response PRS and **B** subjective well-being PRS. **C** Odds ratios for placebo response in the clinical test sample for clinically assessed response PRS. **D** Odds ratios for placebo response and vortioxetine response in the clinical test sample for subjective well-being PRS.

PRS for clinically assessed response, MDD, and subjective well-being were nominally associated with placebo response from several outcome measures (emp. *p*-value < 0.05, Table 2). Higher clinically assessed response PRS was nominally associated with a better placebo response (CPFQ and PDQ improvement: pT = 0.1, beta (SE) = 4.82 (1.54), emp. *p*-value = 0.004), see Fig. 4A. To recapitulate, the most significant association for vortioxetine response in the clinical test sample was also found for clinically assessed response PRS (Table 2, Fig. 2). Furthermore, higher MDD PRS was nominally associated with better placebo response (HAM-A SA improvement pT = 0.00005, beta (SE) = −0.54 (0.19), emp. *p*-value = 0.011). In comparison, higher MDD PRS were suggestive of better vortioxetine response in the clinical test sample in four out of six response measures, see Supplemental Material Fig. S8. Finally, higher subjective well-being PRS showed nominal associations with worse placebo response in the following measures; MADRS improvement (pT = 0.001, beta (SE) = −1.45(0.50), emp. *p*-value = 0.031), MADRS-6 improvement (pT = 0.001, beta (SE) = −0.97(0.34), emp. *p*-value = 0.033) and HAM-A SA improvement (pT = 0.005, beta (SE) = −0.57(0.20), emp. *p*-value = 0.028). Interestingly, the subjective well-being PRS for patients treated with vortioxetine in the clinical test sample consistently showed the opposite trend, where higher PRS was associated with better treatment response, see Fig. 4B, D.

## Discussion

In this study, we collected genetic and clinical data from seven randomized, double-blind, placebo-controlled clinical trials evaluating the efficacy of vortioxetine with a positive outcome. With this clinical test sample and a self-reported test sample with vortioxetine-treated patients, we investigated PRS association with vortioxetine and placebo response assessed using clinical scales, clinical sub-scales and self-reports.

There were no significant associations after Bonferroni correction in our clinical test sample. Clinically assessed response PRS was nominally associated with vortioxetine improvement in anxiety symptoms in our clinical test sample. We observed a stronger association when assessing clinical vortioxetine treatment response on the HAM-A PA sub-scale, compared to total HAM-A score. In the self-reported treatment sample, we found that higher PRS for schizophrenia were associated with poorer self-reported response. Furthermore, we found that higher subjective well-being PRS and lower depression PRS showed nominal associations with higher placebo-response.

In our study, the best predictor of clinically assessed treatment response in patients treated with vortioxetine was the clinically assessed response PRS derived from a GWAS summary statistics base with a sample size of 5218 individuals [10]. The self-reported responders PRS [17] showed no association with the clinically assessed vortioxetine patients even though this PRS was derived from a base GWAS with a larger sample size (12,537 individuals). In a recently published study, PRS for clinically assessed remission showed nominal association with clinically assessed treatment response [10]. Interestingly, no associations were observed between PRS for clinically assessed remission in a cohort with self-reported response and a cohort with treatment-resistant depression defined as individuals that were prescribed at least one antidepressants [10]. This indicates that a PRS for treatment response can be used to predict treatment response.

In the self-reported test sample, we report a significant association between higher PRS for schizophrenia and worse self-reported vortioxetine treatment response. This is in line with previous findings [10, 12, 17].

The fact that we did not observe any associations between the clinically assessed vortioxetine response and disease or symptom trait PRSs based on large-scale GWAS summary statistics (N ranges from 55,374 to 500,199) underlines the gain of power when utilizing a treatment response PRS predictor more correlated to the target, despite the lower power (*N* = 5218), see power calculations for a range of correlations in Supplementary Material B. While exploring treatment response association with disease PRSs can contribute with important learnings, we need more high-quality treatment response data to build useful, predictive models. We believe that, as larger GWAS summary statistics assessing antidepressant treatment responses evolve, they will provide more relevant predictors of treatment response in clinical trials. Hopefully, in the long run, they can mitigate treatment strategies so that patients more rapidly can receive treatment with the highest chance of success.

To the best of our knowledge, this is the first time placebo response is genetically evaluated in a PRS context for patients with depression from clinical trials. For patients treated with placebo, a higher PRS for subjective well-being was nominally associated with poorer placebo response measured by MADRS, MADRS-6, and HAM-A SA. We also observed that a high PRS for MDD was nominally associated with better placebo response (CPFQ & PDQ improvement), consistent with the fact that subjective well-being is negatively genetic correlated med MDD [37]. This is in line with previous studies where higher PRS for MDD [12, 14–16] have shown nominal associations with worse treatment response. The nominal PRS associations to placebo repones should be further explored beyond the current paper, as the identification of genetic variables predictive of placebo response can be useful in screening out high placebo responders from clinical trials and help improve the prediction of true treatment response.

We included sub-scales such as HAM-A SA and HAM-A PA in our analysis, which assess a subset of symptoms within the entire scale. Sub-scales can potentially enhance detection power by investigating more clinically intermediate response outcomes and help point in the direction of which symptoms are driving the signal. Indeed, we observed that the association between PRS for clinically assessed response and treatment response to vortioxetine measured by the HAM-A scale were driven by the sub-scale HAM-A PA. Our results also indicate that using sub-scales can help identify weaker associations which was not found when association with the entire scale was tested. As an example, for the placebo-treated patients both PRSs for MDD and subjective well-being were nominally associated with the sub-scale HAM-A SA but not the entire HAM-A scale.

There are limitations to our study. Despite having collected a phenotypically deep, homogeneous clinical test sample, the results underline the need for larger sample sizes. Power calculations showed that the sample size was adequate for most of the association tests, see section Supplementary Material B. For the PRS associations with an empirical *p*-value < 0.05, the variance explained were in the range 1.2–5.3%. This is in line with previous studies investigating PRS associations with antidepressant response [13, 15, 19, 22, 23]. The low treatment response variance explained by PRS might be due to inadequate sample sizes of our base GWAS, error in the effect size estimates, and inevitable differences between the base and test samples. In addition, PRSs were calculated using an additive model on common genetic variants. Thus, we have not explored whether treatment response is affected by rare variants or other potential interactions with common variants. The amount of variance explained at the current stage is limiting the interpretation on the individual’s level and the clinical utility. Furthermore, in the current study only the *p*-values have been adjusted. The variance explained by the PRSs might be affected by overfitting, as the variance explained is unadjusted. Consequently, larger samples are needed to replicate these findings, so that analyses like these can identify robust relationships between PRS and treatment response.

In summary, our results indicate that a clinical PRS for treatment response has the best potential as a predictor of clinical treatment response in several response measures. This is the first study revealing nominal associations between PRSs and placebo response. Our findings indicate that the inclusion of a placebo group, different clinical scales, and sub-scales can help identify the genetic underpinnings specific to antidepressant response. With these differences in polygenic predictors, the study highlights important aspects of clinical and observational treatment response. Importantly, the variance explained by the PRS associations remains low, which limits clinical application and the utility of prediction on the individual level. The next steps are to obtain more well-powered and phenotypically similar GWAS bases to reveal the full potential of PRSs association with antidepressant treatment and placebo response. Finally, this study provides a useful data resource for future clinical genetic research.

## Supplementary information

Supplementary material

Supplementary material C

## Data Availability

GWAS summary statistics will be shared upon request to the corresponding author.
