# Supplementary material for: Polygenic heterogeneity in antidepressant treatment and placebo response
Source: Transl Psychiatry. 2022 Oct 29;12:456. doi: 10.1038/s41398-022-02221-4 (PMC9617908; doi:10.1038/s41398-022-02221-4)
Supplement: Supplementary file 1 — Supplementary material [file 41398_2022_2221_MOESM1_ESM.docx]

## Supplementary Material A

### Montgomery and Åsberg Depression Rating Scale (MADRS)

The MADRS^1^ is a 10-item scale designed to assess the severity of depression. Items in the scale assess the following symptoms: apparent sadness, reported sadness, inner tension, reduced sleep, reduced appetite, concentration difficulties, lassitude, inability to feel, pessimistic thoughts and suicidal thoughts. Each item is rated from 0 (no symptom) to 6 (severe symptom) which gives a total score that ranges from 0-60.

MADRS-6 is a sub-scale of depression that focuses on what is recognized to be the core symptoms. These are: apparent sadness, reported sadness, inner tension, lassitude, inability to feel, and pessimistic thoughts. The total score ranges from 0-36.

### Hamilton Anxiety Rating Scale (HAM-A)

HAM-A^2^ is a 14-item scale designed to assess severity of anxiety. Items assess the following symptoms: anxious mood, tension, fear, insomnia, intellectual (cognitive) symptoms, depressed mood, somatic (sensory), cardiovascular, respiratory, gastrointestinal, genitourinary, autonomic, somatic (muscular) symptoms and behavior at interview. Each item is rated from from 0 (absent) to 4 (maximum severity) leaving a total score in the range from 0-56.

HAM-A Psychic Anxiety (PA) is a sub-scale of HAM-A that assess the severity of psychic anxiety. These are: anxious mood, tension, fear, insomnia, intellectual (cognitive) symptoms, depressed mood, behaviour at interview. The total score ranges from 0-28.

HAM-A Somatic Anxiety (SA) is a sub-scale of HAM-A that assess the severity of somatic anxiety. These are: somatic (sensory), cardiovascular, respiratory, gastrointestinal, genitourinary, autonomic and somatic (muscular) symptoms. The total score ranges from 0-28.

### Perceived deficits questionnaire (PDQ)

The PDQ^3^ is a 20-item, patient-rated scale designed to assess cognitive impairment/dysfunction. The PDQ can be divided into four subscales each consisting of 5-items: Attention/Concentration, Retrospective memory, Prospective memory and Planning/Organization. Each item is rated from 0 (*never*) to 4 (*almost always*), providing a total score in the range 0-80.

### Massachusetts General Hospital Cognitive and Physical Functioning Questionnaire (CPFQ)

Questionnaire CPFQ^4^ is a 7-item, patient-rated scale designed to measure cognitive and executive dysfunction. The items are: Alertness, motivation, attention, memory, lethargy, lexical access and mental acuity. Each item is rated from 1 (greater than normal) to 6 (totally absent), leaving a total score of 42.

## Supplementary Material B

# Association testing

In the clinical test sample Plink v2 was used to test for genetic association for each of the 6 measures of placebo and vortioxetine response using a linear model and adjusting for sex, age and PC1-PC8. For the Vortioxetine group dose was also included as a covariate.

# SNP heritability (h^2^) and genetic correlations in clinical test sample

In the clinical test sample SNP h^2^ of the 6 placebo and vortioxetine response outcomes were calculated from individual-level data using GREML in the genome-wide complex trait analysis (GCTA)^5^ software and from summary statistics using LD Score regression (LDSC)^6^. In GRML the basic settings and 8 PCs were used. When applying LDSC we constrained the regression intercept to 1 and used LD scores from Europeans of the 1000 genomes project that can be downloaded from the LDSC website. Furthermore, LDSC was used to calculate pairwise genetic correlations between the 6 vortioxetine and placebo response outcomes and the 9 GWAS summary statistics. Cross-trait intercepts were constrained to 1 for all pairs without patient overlap and 0 for pairs with full patient overlap

SNP heritability was estimated using LDSC and GCTA for the six vortioxetine and placebo response outcomes in the clinical test sample. SNP h^2^ estimates were not significantly different from zero. Additionally, genetic correlations between vortioxetine and placebo response outcomes to the nine external GWAS summary statistics were not significant. Figures visualizing SNP-heritability estimates and genetic correlations are in Supplementary Material Figure S1-S7.

# Power calculations

Power to detect association between PRSs and response outcomes in samples with different potential sample sizes were estimated using AVENGEME^7^. Since power estimation is dependent on the power of the external GWAS summary statistics, power was calculated using a low-powered summary statistic, clinically assessed response (N=5,218), and a higher-powered summary statistic, MDD (N= 807,553). SNP h^2^ and prevalence for the external GWAS summary statistics were taken from the literature. SNP h^2^ of placebo and vortioxetine response is unknown therefore power was calculated for a SNP h^2^ of 5 %, 10 %, 20 % and 40 %. Similarly, correlation between the external GWAS summary statistics and response to placebo and vortioxetine is unknown, hence power was calculated for a range of correlations (0.1, 0.2, 0.4, 0.8, 1). All parameters used to conduct power calculations are listed in Table S1.

*Table S1 Parameters used when estimating power to conduct PRS analysis.*

| External GWAS | Base N | Base SNP h^2^ | pT | Sample prevalence | Population prevalence | Target SNP h^2^ | Correlation range |
| --- | --- | --- | --- | --- | --- | --- | --- |
| MDD | 500199 | 0.07 | 0.00005 | 0.34 | 0.1 | 0.05,0.1,0.2,0.4 | 0.1,0.2,0.4,0.8 |
| Clinically assessed response | 5218 | 0.13 | 0.0001 | - | - | 0.05,0.1,0.2,0.4 | 0.4,0.6,0.8,1 |

Power calculations for PRS analysis were conducted for different potential sample sizes and for external GWAS summary statistics for clinically assessed response and for MDD, see Supplementary Material Figure S10 and S11. Association testing between a MDD PRS and a placebo or vortioxetine response outcome requires a sample size of > 500 to obtain power > 80% (81,19%), given a heritability of 10% for the response outcome and a correlation of 40%. When conducting power calculations for clinically assessed response PRS and assuming the same parameters as before, the sample size again had to be > 500 to obtain power > 80% (81,36%).

# Sensitivity analysis

We performed a sensitivity analysis on all of our main PRS association results from the clinical sample (see Table 2), in which we considered whether PRS association to treatment response could be induced by an association with disorder severity at baseline. We tested this, since high baseline scores have the potential of showing a larger absolute change in score from baseline. Firstly, we did not observe any significant correlation between baseline scores and change from baseline score in any of the given symptom measures, which suggested that baseline scores in this particular dataset did not affect our main phenotype in the statistical model. Secondly, we ran all PRS association tests given in Table 2 with baseline disease score as an additional covariate in the models and using the same pT values for each test, as given in table 2. The addition of a baseline disease score did not cause any major changes in the results, evaluated by beta coefficient and p-value (see Table S2).

References

[1] Montgomery SA, Asberg M. A new depression scale designed to be sensitive to change. *Br J Psychiatry* 1979. doi:10.1192/bjp.134.4.382.

[2] Hamilton M. The assessment of anxiety states by rating. Br J Med Psychol. 1959;32:50–5.

[3] Sullivan MJL, Edgley K, Dehoux E. A survey of multiple sclerosis. Part 1: Perceived cognitive problems and compensatory strategy use. Can J Rehabil. 1990;4:99–105.

[4] Fava M, Iosifescu DV, Pedrelli P, Baer L. Reliability and validity of the Massachusetts General Hospital Cognitive and Physical Functioning Questionnaire. Psychother Psychosom. 2009;78:91–97.

[5] Yang J, Benyamin B, McEvoy BP, Gordon S, Henders AK, Nyholt DR, et al. Common SNPs explain a large proportion of the heritability for human height. Nat Genet. 2010;42:565–9.

[6] Bulik-Sullivan BK, Loh PR, Finucane HK, Ripke S, Yang J, Patterson N, et al. LD score regression distinguishes confounding from polygenicity in genome-wide association studies. Nat Genet. 2015;47:291–5.

[7] Dudbridge F. Power and predictive accuracy of polygenic risk scores. PLoS Genet. 2013;9:1003348.

*Tabel S2*

| *PRS associations from Table 2* | | | |  |  |  |  |  |  |  |
| --- | --- | --- | --- | --- | --- | --- | --- | --- | --- | --- |
| **PRS** | **Response measure** | **N** | **Best P_T_** | **N SNP** | **PRS R^2^** | **Full R^2^** | **Beta** | **SE** | **Emp. p** | **Unadjusted p** |
| **Clinical test sample – vortioxetine response** | |  |  |  |  |  |  |  |  |  |
| Clinically assessed response | HAM-A improvement | 758 | 0.0001 | 87 | 0.012 | 0.122 | 0.82 | 0.28 | 0.026 | 0.0033 |
| Clinically assessed response | HAM-A PA improvement | 758 | 0.0001 | 87 | 0.014 | 0.080 | 0.58 | 0.18 | 0.014 | 0.0014 |
| **Clinical test sample – placebo response** | |  |  |  |  |  |  |  |  |  |
| MDD | HAM-A SA improvement | 384 | 0.00005 | 730 | 0.022 | 0.056 | 0.54 | 0.19 | 0.011 | 0.0039 |
| Clinically assessed response | CPFQ & PDQ improvement | 191 | 0,1 | 31466 | 0.053 | 0.088 | 4.82 | 1.54 | 0.004 | 0.0020 |
| Subjective well-being | MADRS improvement | 441 | 0.001 | 834 | 0.019 | 0.043 | -1.45 | 0.50 | 0.031 | 0.0041 |
| Subjective well-being | MADRS-6 improvement | 441 | 0.001 | 834 | 0.019 | 0.042 | -0.97 | 0.34 | 0.033 | 0.0044 |
| Subjective well-being | HAM-A SA improvement | 384 | 0.005 | 2806 | 0.023 | 0.056 | -0.57 | 0.20 | 0.028 | 0.0036 |
| **Self-reported test sample – vortioxetine response** | |  |  |  |  |  |  |  |  |  |
| Schizophrenia | Self-reported response | 742 | 0.0001 | 2325 | 0.036 | 0.080 | -0.283 | 0.058 | 0.0001 |  |
|  |  |  |  |  |  |  |  |  |  |  |
|  |  |  |  |  |  |  |  |  |  |  |
| *PRS association with baseline disease score as covariate* | | | | | | | |  |  |  |
| **PRS** | **Response measure** | **N** | **Best P_T_** | **N SNP** | **PRS R^2^** | **Full R^2^** | **Beta** | **SE** | **Emp. p** | **Unadjusted p** |
| **Clinical test sample – vortioxetine response** | |  |  |  |  |  |  |  |  |  |
| Clinically assessed response | HAM-A improvement | 758 | 0.0001 | 87 | 0.012 | 0.122 | 0.82 | 0.28 | 0.007 | 0.0033 |
| Clinically assessed response | HAM-A PA improvement | 758 | 0.0001 | 87 | 0.014 | 0.078 | 0.58 | 0.18 | 0.004 | 0.0040 |
| **Clinical test sample – placebo response** | |  |  |  |  |  |  |  |  |  |
| MDD | HAM-A SA improvement | 384 | 0,00005 | 730 | 0.022 | 0.056 | 0.54 | 0.19 | 0.007 | 0.0040 |
| Clinically assessed response | CPFQ & PDQ improvement |  |  |  |  |  |  |  |  |  |
| Subjective well-being | MADRS improvement | 441 | 0.001 | 834 | 0.019 | 0.043 | - 1.45 | 0.50 | 0.008 | 0.0041 |
| Subjective well-being | MADRS-6 improvement | 441 | 0.001 | 834 | 0.019 | 0.042 | - 0.97 | 0.34 | 0.009 | 0.0044 |
| Subjective well-being | HAM-A SA improvement | 384 | 0.005 | 2806 | 0.022 | 0.056 | - 0.57 | 0.20 | 0.006 | 0.0036 |
| **Self-reported test sample – vortioxetine response** | |  |  |  |  |  |  |  |  |  |
| Schizophrenia | Self-reported response |  |  |  |  |  |  |  |  |  |

Note: PRS = Polygenic risk score; MDD = Major depressive disorder; ; *MADRS: Montgomery-Åsberg Depression Rating Scale;* MADRS-6; sub-scale of MADRS that focuses on the core symptoms of depression; *HAM-A: Hamilton Anxiety Rating Scale; PDQ: Perceived deficits questionnaire; CPFQ: Massachusetts General Hospital cognitive and physical functioning questionnaire*; N : no. of patients in association test; Best P_T_: Best P-value threshold as defined pr PRSice2; N SNP: no. of SNPs for bets P_T_; PRS R^2^: variance explained by the PRS; Full R^2^: variance explained by the full model (including covariates); Beta: estimated coefficient; SE: standard error; P: P-value; Emp. p: Empirical p-values, *: significant following Bonferroni correction.

## Supplementary Material Figures

*
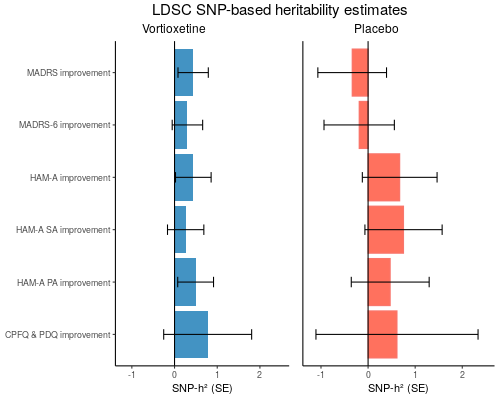
*

*Figure S1 SNP-based heritability estimates for change from baseline in different scales and sub-scales for patients treated with vortioxetine or placebo. The heritability estimates are obtained using LDSC and the intercepts were constrained to 1. The error bars are SE.*


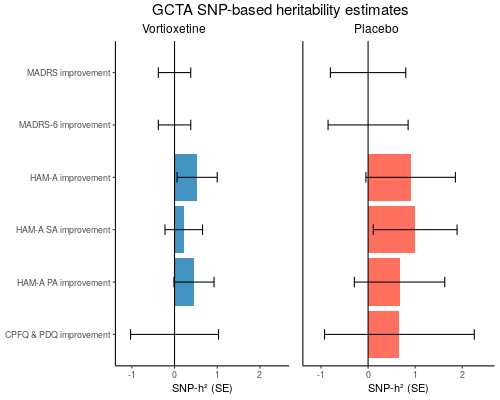


*Figure S2 SNP-based heritability estimates for change from baseline in different scales and sub-scales for patients treated with vortioxetine or placebo. The heritability estimates are obtained using GCTA. The error bars are SE.*


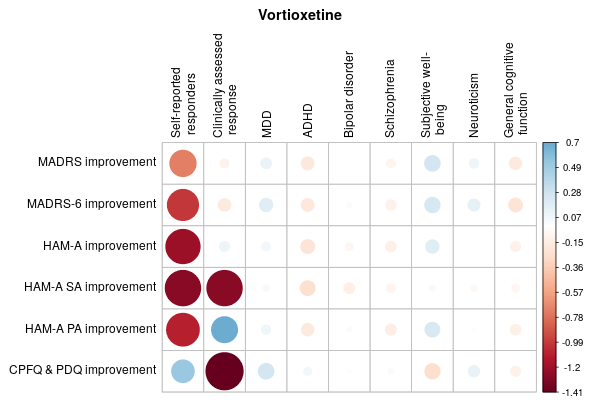


*Figure S3 Genetic correlations between 6 different measures of treatment response outcome in patients treated with vortioxetine and publicly available GWAS summary statistics estimated using LDSC. All regression intercepts and cross-trait intercepts are to 1. None of the correlations had a p-value < 0.05.*

*
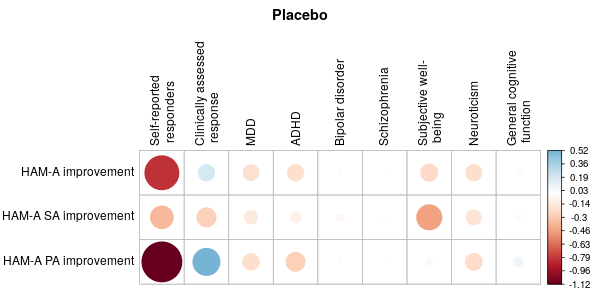
*

*Figure S4 Genetic correlations between 3 different measures of treatment response outcome in patients treated with placebo and publicly available GWAS summary statistics estimated using LDSC. All regression intercepts and cross-trait intercepts are to 1. None of the correlations had a p-value < 0.05. The treatment response outcomes cognition, MADRS and MADRS-6 are not included since LDSC were not able to calculate correlation for these.*


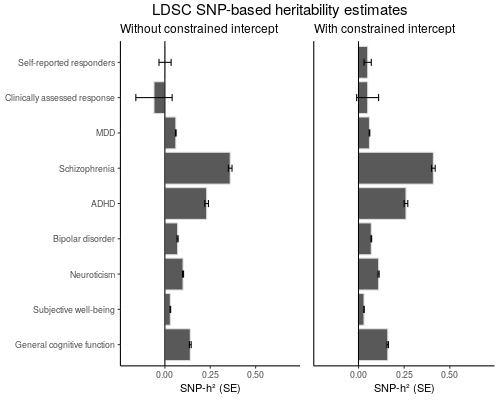


*Figure S5 SNP-based heritability estimates for publicly available GWAS summary statistics. The heritability estimates are obtained using LDSC with and without intercept constrained to 1. The error bars are SE.*

*
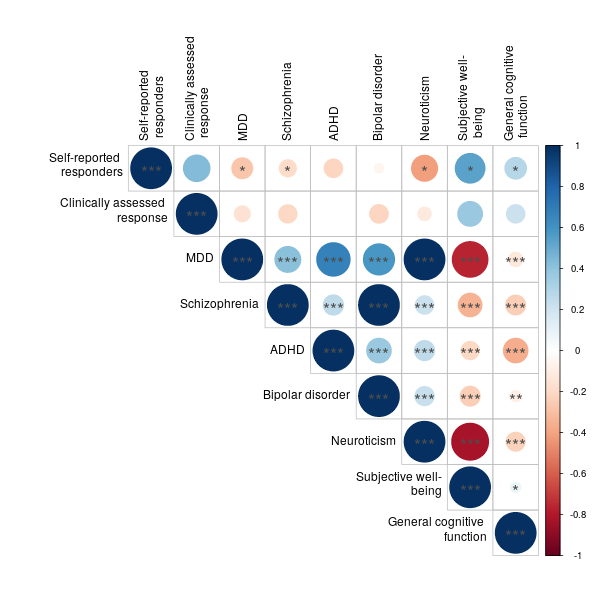
*

*Figure S6 Genetic correlations between publicly available GWAS summary statistics estimated using LDSC with intercept constrained to 1 all phenotypes and cross-trait intercept constrained to 1 for all pairs except for pgcPer vs. pgcRem and ssri vs. ssrB for which the cross-trait intercept is set to 0 since they are based on the same patients. *=p<0.05, **=p<0.001, ***=p<0.00000001.*


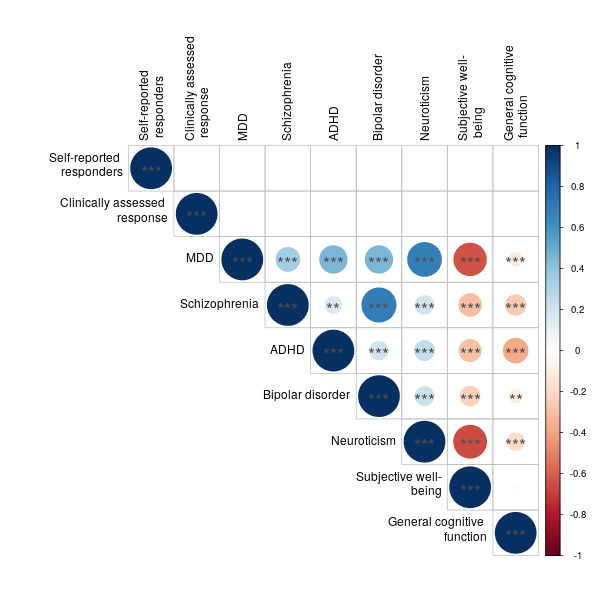


*Figure S7 Genetic correlations between publicly available GWAS summary statistics estimated using LDSC without constrained intercepts. *=p<0.05, **=p<0.001, ***=p<0.00000001.*

##
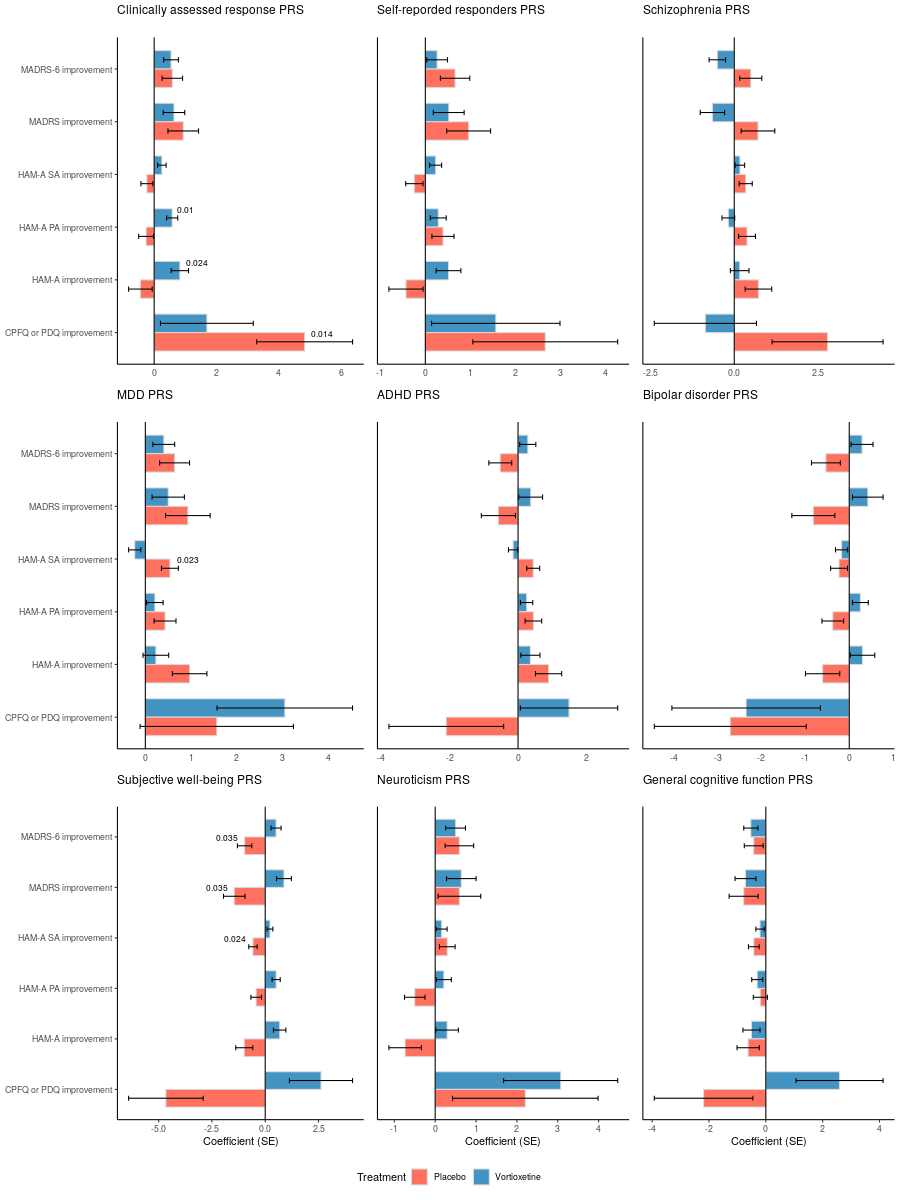


*Figure S8 Bar plot showing the coefficients for all placebo and vortioxetine response outcome measures association with PRSs for antidepressant response, psychiatric disorders, and symptom traits in the clinical test sample. The error-bars are standard errors and a number above the error-bars are the empirical p-value for associations with an empirical p-value <0.05.*


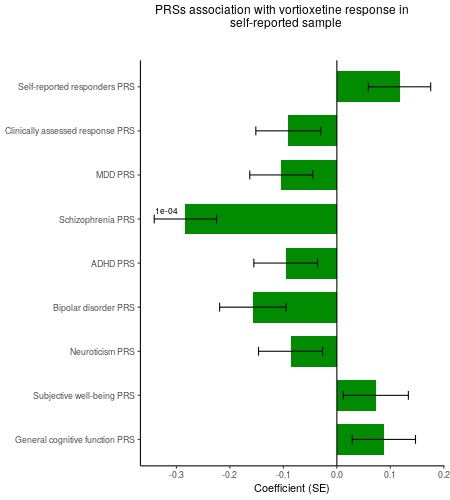


*Figure S9 Association between vortioxetine response in the self-reported sample and PRSs. The error-bars are standard errors and a number above the error-bars are the empirical p-value for associations with an empirical p-value <0.05.*


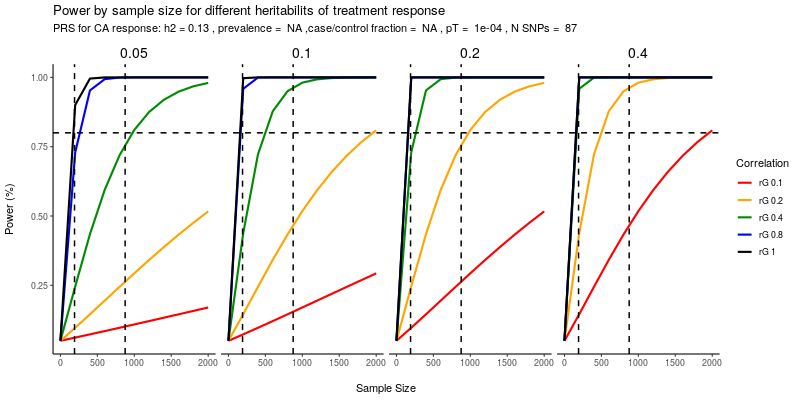


*Figure S10 Estimated power (y-axis) for association between clinically assessed (CA) response PRSs and response outcomes of different sample sizes (x-axis) obtained using AVENGEME. SNP h^2^ of placebo and vortioxetine response is unknown therefore power was calculated for a SNP h^2^ of 0.5, 0.1, 0.2 and 0.4. Likewise, correlation between the external GWAS summary statistics and response to placebo and vortioxetine is unknown, hence power was calculated for a range of correlations (rG: 0.1, 0.2, 0.4, 0.8, 1). The vertical lines outline the range of the sample size of the placebo and vortioxetine response outcomes which were 191 (placebo response outcome PDQ & CPFQ improvement) to 876 (vortioxetine response outcomes MADRS improvement and MADRS-6 improvement).*


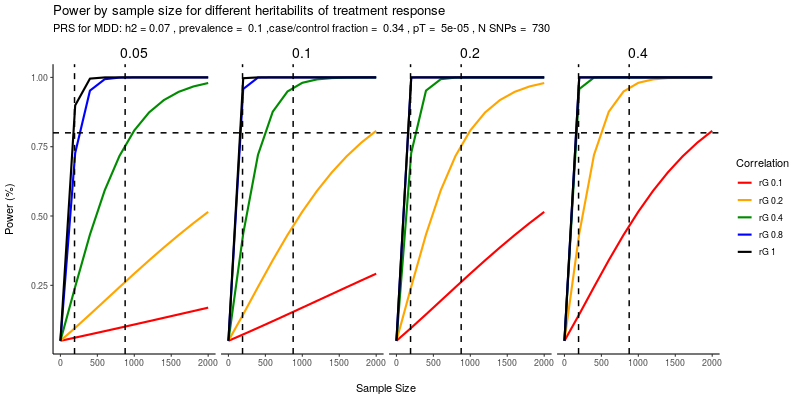


*Figure S11 Estimated power (y-axis) for association between MDD PRSs and response outcomes of different sample sizes (x-axis) obtained using AVENGEME. SNP h^2^ of placebo and vortioxetine response is unknown therefore power was calculated for a SNP h^2^ of 0.5, 0.1, 0.2 and 0.4. Likewise, correlation between the external GWAS summary statistics and response to placebo and vortioxetine is unknown, hence power was calculated for a range of correlations (rG: 0.1, 0.2, 0.4, 0.8, 1). The vertical lines outline the range of the sample size of the placebo and vortioxetine response outcomes which were 191 (placebo response outcome PDQ & CPFQ improvement) to 876 (vortioxetine response outcomes MADRS improvement and MADRS-6 improvement).*

## Supplementary Material Tables

*Table S3 Summary of base GWAS summary statistics*

| Trait | #Cases | Total | #GWAS loci | Population prevalence | LDSC estimated intercept | SNP heritability* | SNP heritability (fixed intercept) |
| --- | --- | --- | --- | --- | --- | --- | --- |
| MDD ^32^ | 170756 | 500199 | 102 | 10% | 1.00 (0.01) | 0.06 (0.002) | 0.06 (0.002) |
| BD ^33^ | 41917 | 413466 | 64 | 1% | 1.02 (0.01) | 0.07 (0.003) | 0.07 (0.002) |
| ADHD ^35^ | 19099 | 53293 | 12 | 5% | 1.03 (0.01) | 0.23 (0.01) | 0.26 (0.01) |
| Schizophrenia^34^ | 53386 | 130644 | 287 | 1% | 1.08 (0.02) | 0.36(0.01) | 0.41 (0.01) |
| Subjective well being^36^ | - | 298420 | 3 | - | 1.00 (0.01) | 0.03 (0.002) | 0.03 (0.001) |
| Neuroticism ^37^ | - | 390278 | 136 | - | 1.03 (0.01) | 0.10 (0.003) | 0.11(0.003) |
| Cognition ^38^ | - | 282014 | 148 | - | 1.05 (0.01) | 0.14 (0.005) | 0.16(0.004) |
| Clinically assessed responds ^13^ | - | 5218 | 0 | - | 1.01 (0.01) | -0.06 (0.10) | 0.05 (0.06) |
| Self-reported responders^14^ | 4056 | 12537 | 1 | 32% | 1.01 (0.01) | 0.001 (0.0334) | 0.05 (0.02) |
| Note: The listed numbers might differ from the original studies, since some of the studies only provide a subset of the individuals they use. SNP heritability is estimated using LDSC . * SNP heritability based on LDSC estimated intercept. – indicates that there is no value to report since the trait is continuous. | | | | | | | |

*Table S4 Patient demographic and characteristics at baseline in the clinical test sample*

|  | **Vortioxetine (907)** | | **Placebo (457)** | |
| --- | --- | --- | --- | --- |
|  | N | Mean(SD)/% | N | Mean(SD)/% |
| **SEX (Female)** | 907 | 69.24 % | 457 | 69.80 % |
| **AGE (yr)** | 907 | 45.22 (12.78) | 457 | 44.82 (12.32) |
| **MADRS total score** | 876 | 32.29 (3.83) | 441 | 31.92 (3.63) |
| **MADRS-6 total score** | 876 | 21.04 (2.72) | 441 | 20.78 (2.76) |
| **HAM-A total score** | 758 | 20.96 (6.15) | 384 | 20.44 (6.02) |
| **HAM-A Somatic Anxiety total score** | 758 | 7.21 (4.07) | 384 | 6.99 (4.00) |
| **HAM-A Psychic Anxiety total score** | 758 | 13.75 (2.94) | 384 | 13.45 (2.88) |
| **PDQ total score** | 115 | 41.04 (12.53) | 61 | 39.11 (12.46) |
| **CPFQ total score** | 223 | 30.51 (4.21) | 130 | 29.95 (4.11) |

| *Note: MADRS: Montgomery-Åsberg Depression Rating Scale; HAM-A: Hamilton Anxiety Rating Scale; PDQ: Perceived deficits questionnaire; CPFQ: Massachusetts General Hospital cognitive and physical functioning questionnaire; SD: standard deviation.* | |
| --- | --- |
|  |  |

|  |  |  |  |  |
| --- | --- | --- | --- | --- |

*Table S5: Improvement between baseline and end of treatment in scales and sub-scales for patients treated with placebo or vortioxetine from the clinical test sample.*

| **Phenotype** | **Scale range** | **placebo** | | **Vortioxetine** | |
| --- | --- | --- | --- | --- | --- |
|  |  | **N** | **Mean (SD)** | **N** | **Mean (SD)** |
| **MADRS improvement** | 0-60 | 441 | 12.95 (10.21) | 876 | 17.16 (10.33) |
| **MADRS-6 improvement** | 0-36 | 441 | 8.78 (6.85) | 876 | 11.23 (6.99) |
| **HAM-A improvement** | 0–56 | 384 | 8.36 (7.751) | 758 | 9.89 (7.90) |
| **HAM-A Somatic Anxiety improvement** | 0-28 | 384 | 5.35 (4.72) | 758 | 6.75 (4.97) |
| **HAM-A Psychic Anxiety improvement** | 0-28 | 384 | 3.01 (3.70) | 758 | 3.14 (3.91) |
| **PDQ or CPFQ improvement** | 0-100 % | 191 | 19.64 (22.40) | 338 | 23.67 (26.64) |
| *Note: MADRS: Montgomery-Åsberg Depression Rating Scale; HAM-A: Hamilton Anxiety Rating Scale; PDQ: Perceived deficits*  *questionnire; CPFQ: Massachusetts General Hospital cognitive and physical functioning questionnaire; Scale range: the potential range of the scores; SD: standard deviation.* | | | | | |
